# Exploring the impact of growth mindset and grit on college students’ athletic performance

**DOI:** 10.3389/fpsyg.2025.1718594

**Published:** 2026-01-05

**Authors:** Yijing Chen, Ziwei Wu, Yin Zhou, Feng Luo

**Affiliations:** College of Sports and Health, Huaihua University, Huaihua, China

**Keywords:** 800-meter run, athletic performance, grit, growth mindset, university students

## Abstract

Psychological factors (e.g., mindset and grit) are increasingly recognized as pivotal to endurance performance, yet their joint influence on endurance task in physical education remains under-explored, especially among Chinese university students. This study tested whether growth mindset and grit predict 800-meter run performance among Chinese university students and whether grit mediates the mindset–performance link. Two hundred fifty-four undergraduates (134 men, 120 women; *M* = 19.26, SD = 0.73) completed domain-specific scales for growth mindset and grit (perseverance of effort and consistency of interest), followed by an 800-meter run. Structural equation modelling showed that growth mindset had no direct effect on finish time; instead, its influence was channeled through perseverance of effort (*β* = 0.23, 95% CI [0.07, 0.38]). Consistency of interest was unrelated to performance. The findings provided the culture-specific, behavioral evidence that belief in improvable ability can lead to sustained effort, which in turn influence athletic performance in a single-occasion endurance task. Pedagogical suggestions were proposed based on the findings.

## 1 Introduction

The 800-meter run represents a particularly challenging event, demanding not only physical endurance and cardiovascular fitness but also psychological resilience and sustained motivation (Bachero-Mena et al., 2021; Cooper et al., 2021; Wang et al., 2021). The complexity of this event lies in its requirement for both aerobic capacity and effective pacing strategies, as well as the ability to overcome fatigue and psychological barriers during the race (Haugen et al., 2021; Sun et al., 2024). Consequently, educators and researchers have devoted considerable attention to identifying factors that can enhance students’ performance in such demanding athletic tasks. Traditional approaches have primarily focused on technical skills, physical fitness, data modeling, and physiological functions (DeWeese et al., 2015; Kolot et al., 2025; Zhao et al., 2023b). However, there is a growing recognition of the importance of psychological factors in optimizing athletic outcomes. Among the various psychological constructs that may facilitate improved performance in the 800-meter run, growth mindset and grit have emerged as particularly promising (Frontini et al., 2021; Sigmundsson et al., 2020).

A growth mindset, as conceptualized by Dweck (2006), refers to the belief that abilities and intelligence can be developed through dedication and hard work. This concept originates from Dweck’s broader mindsets framework, which contrasts growth mindset with a fixed mindset, the belief that abilities are innate and unchangeable (Dweck, 2006; Yeager and Dweck, 2012). Empirical research has demonstrated that a growth mindset is associated with enhanced academic achievement and greater persistence in the face of challenges (Yao et al., 2021a), including within athletic contexts (McNeil et al., 2024). Growth mindset has been shown to foster adaptive coping strategies and resilience, both of which are crucial for sustained athletic engagement and performance improvement (Albert et al., 2021; Parada and Verlhiac, 2022). Grit, defined as perseverance and passion for long-term goals (Duckworth et al., 2007), has since become a central construct in the study of achievement and performance. The grit framework emphasizes sustained effort and consistency of interest over time, even in the face of setbacks (Gao et al., 2024). Research indicates that grit is positively related to academic success and athletic achievement, promoting persistence, self-regulation, and goal attainment (Hwang et al., 2018; Moles et al., 2017). Despite these promising findings, there remains a paucity of research examining the interplay between growth mindset, grit, and athletic performance specifically among university students, particularly in the context of endurance events such as the 800-meter run.

This study is situated within the context of Chinese higher education, focusing on how growth mindset and grit influence 800-meter run performance. This research extends the application of growth mindset and grit frameworks to a new cultural and educational setting, addressing gaps in the literature regarding their relevance for Chinese university students. Practically, the findings may deepen our understanding of the psychological determinants of athletic performance and provide evidence-based recommendations for sports education. By elucidating the roles of growth mindset and grit, this study aims to inform interventions that foster psychological resilience and optimize athletic performance of university students.

## 2 Literature review

### 2.1 Growth mindset and athletic performance

The mindsets framework, developed by Dweck (1999, 2006), posits that individuals’ beliefs about the malleability of their abilities significantly influence their motivation, behavior, and achievement. According to this framework, a growth mindset is characterized by the belief that abilities can be cultivated through effort, effective strategies, and input from others, whereas a fixed mindset entails the belief that abilities are static and unchangeable (Dweck, 2006; Yeager and Dweck, 2012). The mindsets framework has been widely applied to educational settings, where it has been shown to shape students’ motivation, responses to challenges, their willingness to persist after setbacks, and their overall academic trajectories (Blackwell et al., 2007; Claro et al., 2016; Yao et al., 2021b). Individuals with a growth mindset are more likely to embrace challenges, persist in the face of difficulties, and view effort as a pathway to mastery (Dweck, 2006). These adaptive beliefs foster resilience and a proactive approach to learning and performance, which are particularly relevant in demanding contexts such as competitive sports.

Within the domain of athletics, the mindsets framework has been employed to investigate a range of outcomes, including motivation, coping with failure, and performance improvement (Zhang and Li, 2025). Theoretically, a growth mindset is thought to enhance athletic performance by promoting adaptive goal-setting, encouraging sustained effort, and facilitating recovery from setbacks (Nilsen et al., 2025; Yao and Zhu, 2024). Empirical studies have supported these propositions. For example, Orvidas et al. (2018) found that a growth mindset significantly enhances individuals’ motivation, frequency, and persistence in exercise by boosting self-efficacy and exercise identity, making it a key psychological mechanism for promoting physical activity. Similarly, Deng et al. (2025) demonstrated that a growth mindset significantly boosts athletes’ competitive motivation by alleviating competitive stress and fulfilling psychological needs, with even stronger effects among elite athletes, thereby offering a clear entry point for sports psychological interventions. These findings suggest that cultivating a growth mindset may be a valuable strategy for enhancing athletic outcomes, particularly in events that require sustained effort and resilience.

Despite these advances, existing research on growth mindset and athletic performance has notable limitations. Much of the literature has focused on professional or elite athletes (e.g., Loftesnes et al., 2021), with relatively little attention given to college student populations, particularly at Chinese universities. Situated in a Confucian-heritage context, Chinese university students are socialized to view ability as perfectible through diligent effort (Lou and Noels, 2019). This unique cultural context may play a role in students’ mindsets and their subsequent influence on athletic performance. Furthermore, prior studies have often concentrated on team sports or skill-based activities (Shamshirian et al., 2021), rather than endurance events like the 800-meter run. These gaps highlight the need for research that examines the role of growth mindset in athletic performance among university students, particularly within the context of endurance running. The present study seeks to address these limitations by investigating the relationship between growth mindset and 800-meter run performance in a sample of Chinese university students.

### 2.2 Grit and athletic performance

Grit, as conceptualized by Duckworth et al. (2007), refers to the perseverance and passion for long-term goals, encompassing both sustained effort and consistency of interests over time. The grit framework posits that individuals who exhibit high levels of grit are more likely to persist in the face of adversity, maintain motivation over extended periods, and ultimately achieve higher levels of success (Eskreis-Winkler et al., 2014). Grit is distinct from related constructs such as self-control or conscientiousness, as it emphasizes long-term stamina and the ability to overcome obstacles in pursuit of personally meaningful objectives (Duckworth et al., 2007). The construct has received significant attention in educational and organizational psychology, where it has been linked to academic achievement, career success, and well-being (Bowman et al., 2015; Rimfeld et al., 2016).

In the context of sports, grit has been investigated as a predictor of a series of factors, including athletic performance, motivation, training persistence, competitive motivation, and career longevity (Gucciardi et al., 2009; Rumbold et al., 2022). Grit can facilitate athletic success by promoting sustained practice, effective goal-setting, and the ability to rebound from failure (Duckworth et al., 2007). Existing studies have supported these assertions. For example, Doorley et al. (2022) found that college athletes higher in grit showed a rapid rebound in subjective training performance the day after a poor practice. Longitudinally, grit was also positively associated with greater accumulated training volume and more effective use of motivational feedback. Likewise, Cormier et al. (2024) found that grit significantly enhances athletes’ perceived sport performance and well-being. These findings underscore the importance of grit as a psychological resource that can enhance athletic outcomes, particularly in endurance events that require prolonged effort and resilience.

However, the literature on grit and athletic performance is not without its limitations. On the one hand, participants in the existing studies have mainly comprised adolescent athletes, professional athletes, and military cadets (Apró et al., 2024), leaving university students largely understudied. On the other hand, studies have predominantly focused on sprint, ball-sport, or strength events (Larkin et al., 2016), rather than endurance disciplines such as the 800-meter run. These gaps suggest a need for further investigation into the role of grit in athletic performance among diverse student populations and across different types of athletic tasks.

### 2.3 The mediating role of grit between growth mindset and athletic performance

Theoretically, a positive association exists between growth mindset and grit. When individuals believe that ability can be enhanced through effort, they interpret setbacks as learning opportunities and become more willing to sustain long-term commitment, the very essence of grit (Duckworth et al., 2007). This contention has been empirically substantiated by empirical studies within the field of educational psychology (Park et al., 2020; Zhao et al., 2018). Within the sport domain, Wang et al. (2018) found that adolescents who more strongly endorse a growth mindset exhibit higher levels of grit and that this belief mediates the relationship between brain structure and grit. Albert et al. (2021) further showed that athletes who regard effort as a pathway for growth and who firmly believe that ability is changeable subsequently display elevated grit.

As growth mindset is positively related to grit, and both constructs have been shown to independently predict athletic performance, it is plausible that grit mediates the relationship between growth mindset and athletic outcomes. Empirical research has begun to explore this mediating role. Liu (2022) reports that English as a foreign language learners who adopt a growth language mindset display grit in sustained effort and enduring interest, which in turn strengthens their integrative academic motivation and ultimately leads to significant gains in English achievement. Similarly, Zhao et al. (2023b) demonstrated that growth mindset enhances junior high school students’ academic delay of gratification, specifically, their capacity to persist with academic tasks despite immediate entertainment temptations, through the mediating role of grit. These studies provide preliminary evidence for the mediating role of grit; however, further research is needed to clarify the mechanisms underlying these relationships, particularly among university students and in the context of endurance events, such as 800-meter run.

### 2.4 The present study

Building on the above research gaps, this study employs a sample of Chinese university students to examine how growth mindset and the two dimensions of grit (i.e., perseverance of effort and consistency of interest) influence 800-meter run performance. To this end, the present research addresses the following two questions:

1. What are the relationships between growth mindset, the two dimensions of grit, and athletic performance among university students?
2. What are the mediating roles of grit between growth mindset and athletic performance?

## 3 Research methods

### 3.1 Research context and participants

We recruited 254 second-year undergraduates from a comprehensive university in southwest China via convenience sampling. The cohort included 134 men (53%) and 120 women (47%), aged 18–21 years (*M* = 19.26, SD = 0.73). Representing majors such as Chinese Language and Literature and Mathematics and Applied Mathematics, all participants were enrolled in the university’s compulsory physical-education program and had been cleared of cardiovascular or musculoskeletal contraindications by the university entrance medical examination. Before data collection, the research team explained the purpose, procedures, and confidentiality to university administrators, instructors, and participants. Under the institutional policy for minimal-risk anonymous surveys, the Research Office of the College of Physical Education and Health, Huaihua University, granted an exemption from ethics review; returning the completed questionnaire was considered implied informed consent. Participants’ names were collected only to link questionnaire data with 800-m test scores and were removed immediately after merging, leaving a fully anonymized dataset for analysis.

### 3.2 Instruments

We employed the 5-point Likert scale (1 = strongly disagree, 5 = strongly agree) to measure students’ growth mindset and grit. A professional translator translated the English version scales into Chinese and then another professional translator translated the scales back into English. No information was lost during this process. The participants responded to the Chinese version scales.

#### 3.2.1 Growth mindset scale

We adapted Dweck’s (1999) “Implicit Theories of Intelligence Scale—Self Form” (Appendix A). Four items measured growth mindset. To fit the present study, every instance of “intelligence” was replaced with “athletic ability.” For example, the original item “You can always substantially change how intelligent you are”was reworded as “You can always substantially change how capable you are in athletic ability.”

#### 3.2.2 Grit scale

The Grit Scale used in this study was the Domain-specific Grit Scale for College Athletic Students developed by Gao et al. (2024). This scale consists of 10 items, covering two dimensions: perseverance of effort (5 items) and consistency of interest (5 items) (Appendix A). Example statements included “When encountering challenges or setbacks in training, I still give my best effort” (Perseverance of Effort) and “I can maintain a strong interest in my sport over a long period” (Consistency of Interest).

#### 3.2.3 Athletic performance measure

The 800-meter run served as the objective performance indicator. Certified track-and-field referees administered the test according to China’s National Student Physical Fitness Standard. Times were recorded to the nearest 0.1 s and converted uniformly into seconds (s). To align the metric with the “higher-is-better” intuition, we first standardized the raw finish-time data and then reversed the sign: athletic performance = −[(time − M) / SD]. Consequently, a one-unit increase in the performance score reflects a finish time one standard deviation faster than the sample mean.

### 3.3 Data collection

Data were collected during in weeks 2 and 3 of the 2025 fall semester. Thirty minutes before testing began, the classroom teacher assembled the participants in the stadium stands. Students scanned a QR code to access the online survey and completed the scales. A research assistant checked each response for completeness and logical consistency on site, providing assistance if necessary. After the survey was confirmed valid, the 800-meter run was administered according to the national standard. To ensure the 800-meter run reflected typical performance, participants were instructed 48 h beforehand to maintain their normal routines and avoid additional high-intensity training.

### 3.4 Data analysis

We first employed SPSS 26.0 to calculate the descriptive statistics, bivariate correlation, and Cronbach’s alpha coefficients for primary variables. We then employed Mplus 8.3 to perform confirmatory factor analysis (CFA) to examine the construct validity of the scales. Convergent validity was assessed via two key indicators: composite reliability (CR) and average variance extracted (AVE). As suggested by Lu et al. (2024), the recommended cutoffs for establishing acceptable convergent validity are AVE value above 0.50 and a CR value greater than 0.70. For discriminant validity, we followed the criterion proposed by Fornell and Larcker (1981) which stipulated that the square root of the AVE for each latent variable must be larger than the correlation coefficients between that variable and all other latent variables in the model. Finally, we employed Mplus 8.3 to construct a structural equation model (SEM) to examine the relationships between primary variables. Specifically, growth mindset was the independent variable, the two dimensions of grit (i.e., perseverance of effort and consistency of interest) were the mediating variables, and running time was the dependent variable. To evaluate the significance of the indirect effect, we used bias-corrected bootstrapping with 5,000 resamples to construct 95% confidence intervals in Mplus 8.3.

For CFA and SEM models, the following modal-data fit indices were examined: the chi - square statistic (*χ*2), comparative fit index (CFI), root-mean-square error of approximation (RMSEA), and standardized root-mean-square residual (SRMR). As per Meyers et al. (2016), the CFI ought to exceed 0.95, with 0.90 being the minimum acceptable threshold. For RMSEA and SRMR, values below 0.06 are ideal, though values ranging from 0.06 to 0.08 are also deemed acceptable.

## 4 Results

### 4.1 Preliminary analysis results

Descriptive statistics and bivariate correlations for the primary variables, including growth mindset, consistency of interest, perseverance of effort, and running time, are presented in Table 1. The skewness and kurtosis values of all four variables were within the range of −1 to +1, a commonly accepted threshold for approximate normality in behavioral research. This indicated that all variables met the normality requirement for subsequent statistical analyses. Bivariate correlation analyses revealed varying patterns of associations among the variables: growth mindset, consistency of interest, and perseverance of effort remained positively correlated with one another (all *p*s < 0.01). For running time, it showed a positive correlation with consistency of interest and perseverance of effort, while its correlation with growth mindset was non-significant. The Cronbach’s alpha coefficients for the growth mindset and grit scales were all above 0.90, confirming the internal consistency (reliability) of the scales.

**Table 1:** Descriptive statistics and bivariate correlation.

Variable	1	2	3	4
Growth mindset	–			
Consistency of interest	0.68**	–		
Perseverance of effort	0.67**	0.83**	–	
Running time	0.08	0.18**	0.21**	–
Mean (SD)	2.50 (1.04)	2.66 (1.11)	2.79 (1.15)	248.31 (40.82)
Skewness	0.54	0.30	0.14	0.60
Kurtosis	0.16	−0.44	−0.59	0.83
α	0.93	0.92	0.96	/

**p < 0.01, *p < 0.05.

### 4.2 CFA results

We then performed the CFA to examine the measurement model of the three latent variables. The fit indices indicated that the model fit the data within acceptable limits: *χ*2 (74) = 221.041, *p* < 0.001, CFI = 0.961, RMSEA = 0.089, and SRMR = 0.035. Perseverance of effort was positively associated with consistency of interest (*r* = 0.83, *p* < 0.001) and growth mindset was positively related to perseverance of effort (*r* = 0.72, *p* < 0.001) and consistency of interest (*r* = 0.73, *p* < 0.001). The factor loadings of all three variables ranged from 0.76 to 0.93.

The AVE values for growth mindset, perseverance of effort, and consistency of interest were 0.76, 0.82, and 0.71, respectively, and the CR values for the three variables were 0.93, 0.96, and 0.92, respectively. All AVE and CR values exceeded the recommended thresholds, confirming good convergent validity of the measurement model. The square root of AVE for growth mindset (0.87) was greater than its correlations with consistency of interest (0.73) and perseverance of effort (0.72); that for consistency of interest (0.84) surpassed its correlations with growth mindset (0.73) and perseverance of effort (0.83); and the square root of AVE for perseverance of effort (0.91) was larger than its correlations with growth mindset (0.72) and consistency of interest (0.83). Collectively, these results fully met Fornell and Larcker’s (1981) criterion, thereby confirming that the measurement model possessed adequate discriminant validity.

### 4.3 SEM results

**Figure 1:**
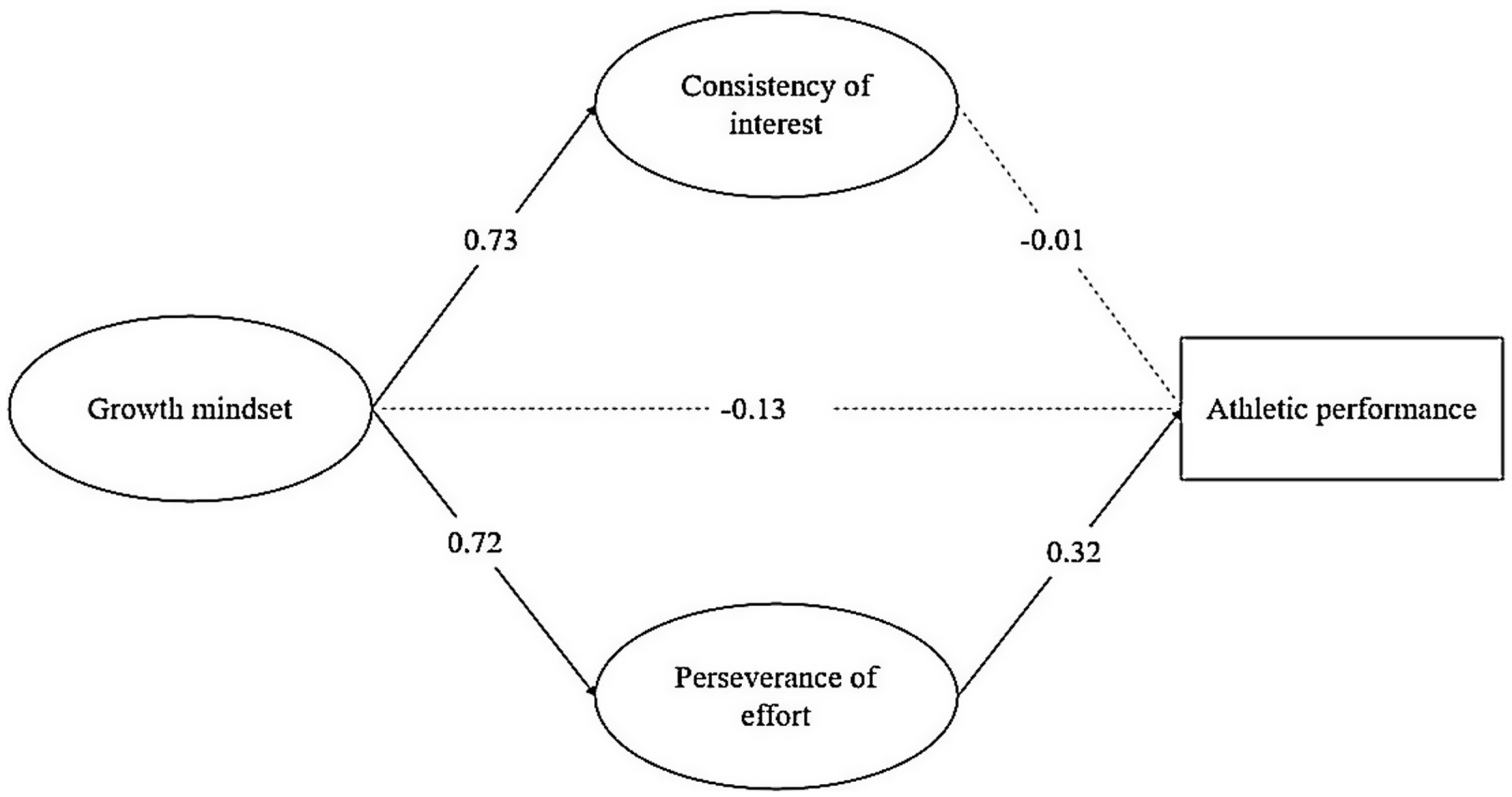
Standardized path coefficients of the model.

Finally, we proceeded to examine the structural model. The fit indices indicated that the model reached acceptable level fit to the data: *χ*2 (85) = 244.636, *p* < 0.001, CFI = 0.958, RMSEA = 0.086, and SRMR = 0.036. As shown in Figure 1, growth mindset was positively associated with both perseverance of effort (*β* = 0.72, *p* < 0.001) and consistency of interest (*β* = 0.73, *p* < 0.001), yet it had no significant relationship with running time (*β* = −0.134, *p* = 0.185). Among the two dimensions of grit, only perseverance of effort was positively related to running time (*β* = 0.32, *p* = 0.04), while consistency of interest was not (*β* = −0.010, *p* = 0.950). We further investigated the indirect effects and found that only the indirect effect of growth mindset on running time via perseverance of effort was significant: *β* = 0.23, 95% CI = [0.07, 0.38].

The R squares for perseverance of effort, consistency of interest, and running time were 0.52, 0.54, and 0.06, respectively (all *p*s < 0.05). The results indicated that approximately 51, 53, and 6% of the variances in the three variables could be explained by the model.

## 5 Discussion

### 5.1 RQ1: interplay of growth mindset, grit, and 800-meter performance

The present study first asked how growth mindset and the two constituent dimensions of grit relate to 800-meter run performance among university students. Three patterns emerged that refine the theoretical mapping outlined in the literature review. First, growth mindset exhibited a strong association with both perseverance of effort and consistency of interest, echoing previous reports in Western adolescent athletes (Wang et al., 2018) and Chinese secondary-school students (Zhao et al., 2018). However, when the two grit dimensions and growth mindset were modelled simultaneously, growth mindset failed to predict 800-meter run performance directly. The null path suggests that simply holding a growth-oriented belief about athletic ability was not enough to improve the 800-meter run performance. These beliefs only became impactful when they were translated into sustained, effortful action, exactly what the perseverance of effort dimension captures (further elaborated in the following section).

Second, although the two grit dimensions were positively associated with 800-meter run performance at the bivariate level, only perseverance of effort emerged as a significant psychological predictor when both dimensions were entered into the structural model simultaneously. This asymmetry corroborates the finding with Canadian university students that the predictive utility of grit on athletic performance resides primarily in the dimension of perseverance of effort rather than consistency of interests (Cormier et al., 2024). One possible explanation is that endurance events such as the 800-meter run are characterized by acute physiological discomfort, particularly in the final 200-meter surge. Athletes who habitually invest sustained effort despite aversive internal states are therefore more likely to maintain pace integrity and optimize finish time. Consistency of interest, despite its long-term benefits (Neroni et al., 2022), may be less pertinent to a single-season fitness test where momentary exertion overrides affective continuity.

Taken together, the findings indicate that among Chinese university students, growth mindset constitutes a facilitative belief system that must be translated into sustained, effortful action to exert an ergogenic effect on 800-meter run performance. Perseverance of effort functions as the operative psychological lever, whereas consistency of interest appears secondary in the context of a discrete, time-trial endurance task.

### 5.2 RQ2: grit as a mediator between growth mindset and 800-meter performance

The second research question asked whether grit mediates the effect of growth mindset on 800-meter run performance. Overall, the mediation findings suggest that growth mindset does not operate as a direct performance catalyst; rather, it functions as a cognitive primer that activates perseverance of effort, which subsequently translates into observable speed improvements. This finding advances the literature in three ways.

First, it provides behavioral evidence for the motivational sequence proposed by Dweck (2006). That is, belief in the malleability of ability fosters a willingness to exert sustained effort in the face of setbacks, which in turn enhances objective achievement. The present data replicate this sequence in a Chinese university sample and extend it to an acute athletic task, complementing previous academic-domain findings (e.g., Liu, 2022; Yao and Zhu, 2024; Zhao et al., 2023a; Zhao et al., 2023b). Second, the specificity of the indirect path to perseverance of effort corroborates the argument that the staying component of grit, rather than the liking component, is the active ingredient in short-cycle performance contexts (Cormier et al., 2024). Besides, the magnitude of the indirect effect may partly reflect culture-specific meaning systems. Confucian cultures prioritize self-improvement and effort-based attributions (Lou and Noels, 2019), leading students to translate growth beliefs into sustained exertion. As illustrated in the previous section, maintaining pace during the final 200-meter surge of an 800-meter run demands continuous self-regulation against physiological aversion; it is therefore conceptually consistent that a trait capturing continued effort functions as a small but significant psychological pathway through which mindset beliefs are translated into slightly quicker finish times.

Third, the absence of an indirect effect via consistency of interest supports suggests that affective stability may be more relevant for longitudinal outcomes than for single-occasion maximal efforts (Neroni et al., 2022). Within the temporal boundary of a compulsory fitness test, momentary exertion outweighs long-term passion, rendering consistency of interest secondary for immediate performance gains.

### 5.3 Pedagogical suggestions

The finding that perseverance of effort, rather than consistency of interest, carries the influence of growth mindset to better 800-meter run performance suggests three straightforward adjustments to everyday university physical education practice. First, instructors can open each endurance unit with a brief “effort-is-improvable” module, for example, a five-minute presentation of evidence on aerobic trainability, followed by students setting a personally demanding yet realistic split-time goal for the upcoming time trial. This procedure may keep the growth belief active and link it to an immediate behavioral target, repeating the cycle at the start of every training block to maintain salience without consuming additional curriculum hours.

Second, teachers may consider structuring workouts that deliberately practice “push-through” moments. Inserting coached final-lap simulations, such as three repetitions of 600 m at 95% race pace with 90-s recovery, allows students to experience and self-regulate the critical 200-meter surge where pace integrity is typically lost. A 30-s written reflection immediately after each repetition (“What strategy helped me hold form when fatigue peaked?”) may reinforce a controllability attribution (Guan et al., 2024) and consolidates the mindset-perseverance-performance sequence observed in our model.

Finally, feedback language should be shifted from outcome or interest based to effort contingent (Dweck, 2006; Yao et al., 2024; Yao et al., 2025). Rather than praising “natural speed” or exhorting students to “love running,” instructors can highlight observable perseverance, such as “Your kick in the last 200 meter shows you maintained cadence under fatigue which is the evidence that aerobic capacity is expanding.” Such comments align with not only the argument that feedback should focus on effort and strategy (Dweck, 1999, 2006) but also with the mediating mechanism observed in the present study, strengthening exercise-specific growth language and providing a clear behavioral cue that students can replicate in subsequent trials. These low-cost, theory-aligned practices should help university students translate abstract beliefs about improvable ability into the sustained, effortful actions that demonstrably improve 800-meter run performance.

## 6 Conclusion

The present study examined whether growth mindset and the two dimensions of grit predict 800-meter run performance among Chinese university students and whether grit mediates the mindset–performance link. The results revealed that growth mindset bore no direct relation to 800-meter run performance; instead, its effect was fully transmitted through perseverance of effort. These findings offer modest, culture-specific evidence for the motivational sequence “malleability belief–sustained effort–athletic achievement” in a one-off endurance task, thereby extending Dweck’s (1999, 2006) framework to physical-education settings and indicating which facet of grit may function as a modest psychological mechanism.

Practically, the results signal that university coaches should pair growth-oriented messaging with effort-contingent drills if they wish to translate psychological beliefs into measurable gains on the track.

There are also several limitations in this study that merit further research. First, the use of convenience sampling within one south-western Chinese university restricts external validity. Replication across multiple provinces and institution types is needed to establish generalizability. Second, the cross-sectional design cannot establish causal ordering or track temporal stability. Future work should employ longitudinal or intervention designs to test whether mindset–perseverance effects accumulate across training macrocycles. Third, the dependent variable was a single maximal test. Researchers could investigate whether consistency of interest becomes predictive when performance is operationalized as accumulated training volume or season-long improvement. Finally, the dependent variable was a single maximal 800-meter test completed during a compulsory fitness session. The result may be influenced by daily form, pacing errors, or short-term motivation rather than stable endurance capacity. Future studies should employ repeated time trials, seasonal best records, or cumulative training volume to align the performance measure with the long-term nature of the psychological constructs examined.

## Data Availability

The raw data supporting the conclusions of this article will be made available by the authors, without undue reservation.

## Appendix A. Instruments

### Growth mindset scale

1. No matter who you are, you can significantly improve your level of athletic ability.
2. You can always substantially change how capable you are in athletic ability.
3. Regardless of your current athletic ability level, you can always change it to a great extent.
4. Even your basic level of athletic ability can be substantially changed.

Adapted from Dweck (1999).

### Grit scale

#### Perseverance of effort

1. When encountering challenges or setbacks in training, I still give my best effort.
2. When training feels uncomfortable or unfamiliar, I still give my best effort to push beyond my current limits.
3. When I receive negative feedback or criticism, I still give my best effort to stay motivated and keep training.
4. When progress is slow or invisible, I still give my best effort and remain patient with the process.
5. When a session becomes mentally tough, I still give my best effort to stay focused and finish what I started.

#### Consistency of interest

1. I consistently stay motivated to train for my sport and rarely skip or cut short my workouts.
2. I stay fully engaged and focused every time I train for my sport.
3. I consistently prioritize training and avoid making excuses, knowing it benefits my performance and long-term success.
4. I follow my training schedule consistently and stay on track with my plan.
5. I find training for my sport enjoyable and prefer to spend my time on it over other activities.

Adapted from Gao et al. (2024).
